# Liver-FDG-uptake augments early PET/CT prognostic value for CD19-targeted CAR-T cell therapy in diffuse large B cell lymphoma

**DOI:** 10.1186/s13550-025-01201-1

**Published:** 2025-03-17

**Authors:** Michael Beck, Viktoria Blumenberg, Veit L. Bücklein, Ralph A. Bundschuh, Dennis C. Harrer, Klaus Hirschbühl, Johannes Jung, Wolfgang G. Kunz, Karin Menhart, Michael Winkelmann, Igor Yakushev, Anna Lena Illert, Markus Eckstein, Simon Völkl, Rainer Claus, Leo Hansmann, Judith S. Hecker, Torsten Kuwert, Andreas Mackensen, Marion Subklewe, Dirk Hellwig, Fabian Müller

**Affiliations:** 1 Department of Nuclear Medicine, University Hospital of Erlangen, Friedrich-Alexander-Universität- Erlangen Nürnberg, Erlangen, Germany; 2 Bavarian Cancer Research Center, Resp. Site (Augsburg, LMU Munich, TUM Munich, Erlangen, Regensburg), Germany; 3 Department of Medicine III, LMU University Hospital, LMU Munich, Munich, Germany; 4 Laboratory for Translational Cancer Immunology, LMU Gene Center, LMU Munich, Munich, Germany; 5 German Cancer Consortium (DKTK), Partner Site Munich, Munich, Germany; 6 Massachusetts General Hospital, Harvard Medical School, Boston, MA USA; 7 Nuclear Medicine, Faculty of Medicine, University Hospital of Augsburg, Augsburg, Germany; 8 Department of Nuclear Medicine, University Hospital Carl Gustav Carus at the TU Dresden, Dresden, Germany; 9 Department of Internal Medicine III, Hematology and Medical Oncology, University Hospital of Regensburg, Regensburg, Germany; 10 Hematology and Oncology, Medical Faculty, University of Augsburg, Augsburg, Germany; 11 Department of Medicine III, School of Medicine and Health, Technical University of Munich (TUM), Munich, Germany; 12 Department of Radiology, LMU University Hospital, LMU Munich, Munich, Germany; 13 Department of Nuclear Medicine, University Hospital Regensburg, Regensburg, Germany; 14 Department of Nuclear Medicine, School of Medicine, TUM University Hospital, Technical University of Munich, Munich, Germany; 15 Department of Pathology, University Hospital of Erlangen, Friedrich-Alexander-Universität- Erlangen Nürnberg, Erlangen, Germany; 16 Department of Internal Medicine 5, Hematology and Oncology, University Hospital Erlangen, Friedrich-Alexander-Universität-Erlangen Nürnberg, Erlangen, Germany; 17 Pathology, Faculty of Medicine, University of Augsburg, Augsburg, Germany; 18 Center for Translational Cancer Research, Technical University of Munich (TUM), TranslaTUM, Munich, Germany

**Keywords:** CAR T cell therapy, DLBCL, FDG PET, Liver-SUV

## Abstract

**Background:**

Despite revolutionary efficacy of CD19-CAR-T cell therapy (CAR-T) in aggressive B cell lymphoma, many patients still relapse mostly early. In early failure, distinct drugs support CAR-T which makes reliable and early prediction of imminent relapse/refractoriness critical. A complete metabolic remission (CR) on Fluor-18-Deoxyglucose (FDG) Positron-Emission-Computed Tomography (PET) 30 days after CAR-T (PET30) strongly predicts progression-free survival (PFS), but still fails in a relevant proportion of patients. We aimed to identify additional routine parameters in PET evaluation to enhance CAR-T response prediction.

**Results:**

Thirty patients with aggressive B cell lymphoma treated with CAR-T were retrospectively analyzed. Pre-CAR-T, LDH was the strongest PFS-predictor also by multivariate analysis. Post-CAR-T, 10 out of 14 patients (71.4%) with PET30-CR remained in disease remission, while 12 out of 16 patients (75%) with incomplete metabolic remission (PET30-nCR) relapsed after CAR-T. 28.6% of patients with PET30-CR ultimately progressed. Change of liver FDG-uptake from baseline to day30 (Delta-Liver-SUV_mean_) was identified as an independent biomarker for response. PET30-nCR and a decrease of Delta-Liver-SUV_mean_ were associated with a high risk of tumor progression (HR 4.79 and 3.99, respectively). The combination of PET30 and Delta-Liver-SUV_mean_ identified patients at very low, at intermediate and at very high risk of relapse (PFS not reached, 7.5 months, 1.5 months, respectively).

**Conclusion:**

Additionally to PET30 metabolic remission, longitudinal metabolic changes in Delta-Liver-SUV_mean_ predicted CAR-T efficiency. Our results may guide early intervention studies aiming to enhance CAR-T particularly in the very high-risk patients.

**Supplementary Information:**

The online version contains supplementary material available at 10.1186/s13550-025-01201-1.

## Introduction

Therapy-refractory or early recurrent diffuse large B cell lymphoma (DLBCL) and transformed follicular lymphoma (tFL) together account for most of the aggressive B cell lymphoma and had dismal prognosis in the pre-CAR-T era [1]. In early second and any later relapse, autologous anti-CD19 chimeric antigen receptor T cell therapy (CAR-T) revolutionized treatment by achieving substantially higher rates of long-term remission than the previous standard of care [2–4]. In studies with extended follow-up, lasting responses following CAR-T were maintained in 60 to 76% of all patients that had achieved complete responses and suggest CAR-T to be a likely curative treatment approach for a subgroup of patients [5, 6].

Generally, established models to predict durable responses to CAR-T distinguish between pre and post CAR-T cell-infusion. Pre-CAR-T, tumor burden defined as tumor volume by contrast-enhanced CT-scan, as total metabolic tumor volume (TMTV) by Positron Emission Computed Tomography (PET)-scans or estimated by serum-Lactate dehydrogenase (LDH) are strong predictors of lasting responses [7–10]. Other patient characteristics at lymphodepletion (LD) before CAR-T infusion that individually are associated with poor performance are extra-nodal disease, Eastern Cooperative Oncology Group Performance Status (ECOG), and response to bridging therapy [10–14]. Only analyzed in small and retrospective cohorts, the local lymphoma immune-microenvironment before CAR-T may also serve as predictor of response [15–17]. Classic combinations of patient characteristics that predict survival in first-line therapy such as the revised international prognostic index (R-IPI) perform poorly in predicting survival after CAR-T. In contrast, the novel international metabolic prognostic index (IMPI) which is based on TMTV, age, and stage at LD performs better [12].

PET imaging is the most precise method to evaluate responses in aggressive lymphoma [18] and is thus the most reliable imaging modality frequently used to predict treatment response at day 30 following CAR-T [19]. However, up to 30% of patients that show a complete metabolic remission (CR) in PET on day 30 (PET30) do not achieve long-term remission (false-negative prediction) and relapse within 12 months from CAR-T cell infusion [19, 20]. Because the reasons for CAR-T failure are diverse and include CD-19 loss, disadvantageous tumor microenvironment, insufficient initial CAR-T cell expansion, or poor CAR-T cell persistence [13, 16, 21], it is not surprising that a single measurement is not enough to reliably predict response early after CAR-T. Successful early prediction may have therapeutic consequence as imminent CAR-T failure can be prevented by early immune modulation using checkpoint inhibitors, -imids, bispecific antibodies or BTK-inhibitors [23–27] which has successfully supported insufficiently working CAR-T cells. However, to reliably identify patients who could potentially benefit from such early intervention, the prediction of CAR-T failure needs further improvement.

There has been some evidence from few DLBCL patients after CAR-T and from a study in Hodgkin’s lymphoma that patients who achieve a lasting remission after therapy show an increase of mean standardized uptake values (SUV_mean_) in spleen or liver (Liver-SUV_mean_) in PET-scans compared with measures before therapy [8, 22]. In line, we hypothesized that a combination of PET30 and change of Liver-SUV_mean_ more reliably predicts early relapse and, as such, identifies patients in need of novel treatment combinations in imminent treatment failure.

## Materials and methods

### Patients and data collection

In this multicenter and retrospective analysis across five Bavarian university hospitals, we aggregated data from patients treated with CD19-CAR-T cells (Tisagenlecleucel (Tisa-Cel), Axicabtagen-Ciloleucel (Axi-Cel), Lisocabtagene Maraleucel (Liso-Cel) and an experimental CD-19-CAR-T product between October 2019 and September 2023.

Patients eligible for analysis had to be treated with CAR-T due to relapsing or therapy-refractory DLBCL or tFL and had undergone a baseline PET scan (BL) prior to the start of CAR-T as well as a PET30 scan after CAR-T. Additionally, a documented progression at any given time or a documented progression-free survival of at least 6 months was required. Overall, 30 patients met the inclusion criteria. LD was done using Fludarabine/Cyclophosphamide. This study was carried out in compliance with the declaration of Helsinki and with the data protection regulations of the Bavarian University Hospital Act and was approved after examination by the local Ethics Committee (24-128Br). Follow-up was defined as time between CAR-T and the last clinical contact or death. Progression-free survival (PFS) was defined as time between CAR-T and progression according to Lugano criteria or disease associated death [18].

### Fluor-18-Deoxyglucose-PET

Fluor-18-Deoxyglucose (FDG)-PETs were performed on dedicated PET/CT systems (GE Healthcare Chicago ILL, USA, and Siemens Healthineers Erlangen, Germany) following international guidelines and internal standards. EARL (EANM Forschungs GmbH) accreditation was available for all involved PET/CT scanners. Visual and quantitative assessment was performed on dedicated workstations using proprietary software at each respective center. Experienced, board certified radiologists and nuclear medicine specialists performed the evaluation. Pathologic metabolically active lesions were classified according to the Deauville Score (DS) and Lugano criteria [18]. A DS ≤ 3 on PET30 was defined as CR, while a DS ≥ 4 was rated as an incomplete metabolic remission (nCR). Standardized Uptake Values (SUV) and Metabolic Tumor Volume (MTV) were measured on a per-lesion basis using isocontour volume of interest (VOI) either manually drawn or placed via an auto-segmentation tool of the software vendor with a predefined threshold of 41% of the lesion SUV_max_ [28]. Liver SUV_mean_ was measured and its change over time determined as Delta-Liver-SUV_mean_ (PET30 Liver-SUV_mean_– BL Liver SUV_mean_) [29]. As second reference region, background activity in the mediastinal blood pool was measured. TMTV was calculated from the sum of all pathological lesions. IMPI was determined using age, Ann Arbor disease stage, and baseline TMTV accordingly to Mikhaeel et al. [12, 30].

### Statistical analysis

Statistical analysis was done using SPSS v28.0.0.0 (IBM Corporation, Armonk, NY, USA). Values are reported as median ± standard deviation with 95% confidence intervals in square brackets. Correlations were estimated using Spearman Rho Test. Tests for differences were used as indicated. Univariate comparison was performed using Mann-Whitney U-test for continuous variables and Pearson chi-square test for non-continuous variables. Multivariate analysis was performed step-wise. Receiver operator characteristics (ROC) were calculated with area under the curve (AUC), and ideal thresholds were identified using the Youden Index. Factors influencing progression-free survival were examined using Kaplan-Meier curves, Log rank tests, and a functional linear Cox regression model as indicated. Statistical significance was assumed at *p* < 0.05.

## Results

### Patient characteristics and variables that predicted outcome

**Fig. 1:**
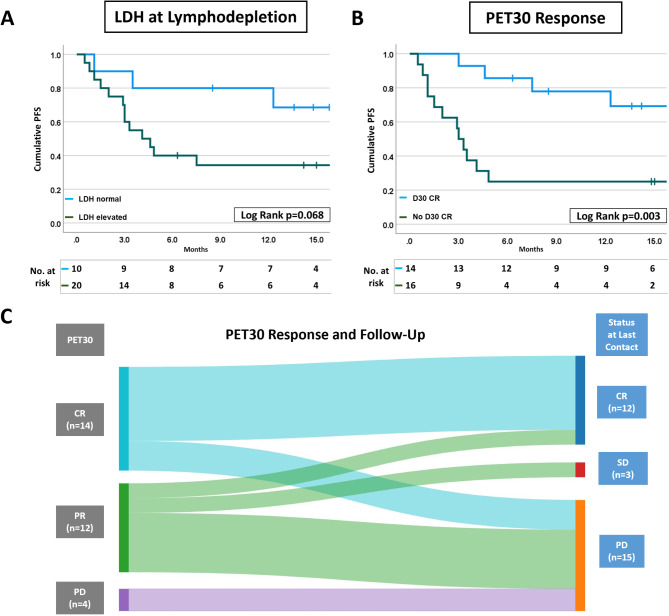
Clinical parameters predicting PFS. Kaplan-Meier statistics for **A** LDH and **B Day 30** PET/CT (PET 30) Response predicting tumor progression after CAR-T. Kaplan-Meier statistics were analyzed by the log-rank test. LDH was determined at lymphodepletion before CAR-T infusion. **C** Sankey Diagram depicting the per-patient development of disease remission status from PET30 to disease outcome at last contact. CD19-CAR-T cell therapy (CAR-T), Lactate Dehydrogenase (LDH), progression free survival (PFS), Total Metabolic tumour volume (TMTV)

Thirty patients were included with a median clinical follow-up of 15.0 ± 8.4 months. PET scans were done at BL (in median 16.5 days before CAR-T; Inter-Quartile Range 36 days) and at day 30 post CAR-T (Median Day 33; Inter-Quartile Range 14 days). Sixteen patients (53.3%) showed tumor progression at a median of 3.0 [1.9–5.0] months after CAR-T and 14 demonstrated long-term disease remission with a median follow-up of 17.7 [14.3–23.3] months. Ten patients had died at data cut-off, of whom eight patients succumbed to disease progression, one died from therapy-related adverse events, and one patient from death by undetermined cause. Elevated LDH at LD was more commonly found in the group of relapsing patients (*p* = 0.019) (Fig. 1A). In line, patients with normal LDH at LD tended to have a better PFS than patients with an LDH above the upper limit of norm (ULN) of 250 U/L (log-rank *p* = 0.068). ROC analysis of LDH as continuous variable determined an optimal cut-off of 272 U/l in our cohort. Patients with an LDH at LD below this threshold showed a significantly longer PFS (Log Rank *p* = 0.002; Suppl. Figure S1). Age, disease stage, extra-nodal disease (Suppl. Figure S1), and IPI (Table 1) did not significantly predict patient groups with distinct PFS.

**Table 1:** Patient characteristics

	Total (*n* = 30)	Progression after CAR-T		Univariate *p*-values*	Multivariate *p*-values^#^
		Yes (*n* = 16)	No (*n* = 14)		
**Sex**					
Female Male	13 17	8 8	5 9	0.484	0.448
**Age**	67.7 ± 9.4	67.7 ± 7.5	65.5 ± 11.4	0.951	0.538
**Disease Type**					
DLBCL tFL	29 1	16 0	13 1	0.467	0.293
**Disease Stage at LD**					
Stage I Stage II Stage III Stage IV	3 4 5 18	1 2 4 9	2 2 1 9	0.571	0.803
**CAR-T Product**					
Tisa-Cel Axi-Cel Liso-Cel Experimental CD19	8 17 4 1	4 8 3 1	4 9 1 0	0.586	0.281
**Bridging Therapy**					
None Chemotherapy Targeted Therapy	5 24 1	2 14 0	3 10 1	0.419	0.795
**Response to Bridging**					
PR SD PD No Data	11 5 8 6	4 5 5	7 0 3	0.055	-
**IPI at LD **					
IPI 1-2 IPI 3–5	11 19	4 12	7 7	0.257	0.193
**LDH at LD (g/dl)**	299.5 ± 201.8	338.5 ± 243.6	237.5 ± 92.4	**0.019**	**0.033**
**CRP at LD (mg/l)**	1.3 ± 39.6	2.5 ± 53.7	0.8 ± 6.1	0.179	0.292
**Ferritin at LD (ng/ml);***n* *= 28*	347.5 ± 381.6	377 ± 344.4	296.0 ± 423.1	0.928	-
**Median Follow Up ** **(Months)**	15.0 ± 8.4				

*p-values were determined by Chi-Square test or by Pearson. # Response to Bridging and Ferritin at LD were excluded from multivariate analysis due to missing values

Abbreviations: Axicabtagen-Ciloleucel (Axi-Cel), CD19-CAR-T cell therapy (CAR-T), Complete remission (CR), C-reactive protein (CRP), Diffuse large B cell lymphoma (DLBCL), International Prognostic Index (IPI), Lisocabtagene Maraleucel (Liso-Cel), Lymphodepletion (LD), partial remission (PR), progressive disease (PD), stable disease (SD), Tisagenlecleucel (Tisa-Cel), transformed follicular lymphoma (tFL)

### PFS prediction based on PET

We then analyzed our cohort for known PET-based predictive markers with a focus on distinguishing pre- and post-CAR-T measurements (Table 2). The BL TMTV in the group of patients with disease progression (mean 82.9 ± 145.4 ml) and in the non-progressive group (28.0 ± 146.0 ml) were not significantly different (*p* = 0.448). ROC analyses identified the optimal threshold for disease progression as a BL TMTV above 7.6 ml (AUC 0.583) in our cohort. However, BL TMTV could not be used to correctly predict tumor progression (*p* = 0.175; Supplementary Figure S1). The IMPI which integrates TMTV, age, and disease stage showed highest discriminative power at a cut-off of 86.4 (AUC 0.571). Low vs. high IMPI could not separate the PFS of the two groups (*p* = 0.254, Supplementary Figure S1).

**Table 2:** Patient grouping based on quantitative and visual PET-scan analysis

	Total	Progression after CAR-T		Univariate *p*-values*	Multivariate *p*-values
		Yes (*n* = 16)	No (*n* = 14)		
**TMTV (ml)**					
BL PET30 Delta TMTV	62.0 ± 143.3 1.5 ± 135.3 –31.3 ± 144.9	82.9 ± 145.4 4.3 ± 173.7 –37.1 ± 159.9	28.0 ± 146.0 0.1 ± 53.4 –27.9 ± 123.4	0.448 0.064 0.697	0.795 0.134 0.261
**IMPI**	85.7 ± 7.4	85.3 ± 7.3	86.1 ± 7.7	0.525	0.723
**PET30 CR**					
Yes (DS ≤ 3) No (DS ≥ 4)	14 16	4 12	10 4	**0.026**	**0.010**
**Delta Liver-SUV**_**mean**_^**$**^	0.1 ± 0.4	-0.1 ± 0.4	0.3 ± 0.3	**0.010**	**0.014**
**Delta Blood Pool-SUV**_**mean**_^**#**^	0.050 ± 0.31	0.00 ± 0.34	0.18 ± 0.23	0.052	0.054

*p-values were determined by Chi-Square test or by Pearson

^$^Delta Liver-SUV_mean_= Liver-SUV_mean_ at PET30 - Liver-SUV_mean_ at BL

#Delta Blood Pool-SUV_mean_= Blood Pool-SUV_mean_ at PET30– Blood Pool-SUV_mean_ at BL

Abbreviations: CD19-CAR-T cell therapy (CAR-T), Complete remission (CR), Deauville Score (DS), PET/CT at Baseline (BL) PET/CT on Day 30 after CAR-T (PET30), International Metabolic Prognostic Index (IMPI), standardized uptake values (SUV), total metabolic tumour volume (TMTV).

We then correlated post-CAR-T PET-measurements with patient survival. ROC analysis of post-CAR-T TMTV in PET30 showed best prediction at a low cut-off volume of 0.4 cm³ (AUC 0.701). Accordingly, PET30-CR defined as a residual uptake in lymphoma lesions equal to or below the liver uptake (DS 1–3) strongly predicted PFS (*p* = 0.003) with a median PFS of 3.0 [2.2–3.8] months in patients with residual metabolic disease compared with a not yet reached PFS in patients with PET30-CR (Fig. 1B**)**. Visualization of patient journeys using a Sankey Diagram supported that most patients with PET30-CR remained in CR (true negative) at last follow-up while the majority of patients with active disease in PET30 eventually progressed (true-positive; Fig. 1C). However, 4 patients were predicted incorrectly as positive (false-positive rate 25.0%) as they are alive without relapse in the follow-up and 4 as false-negative (28.6%) despite CR in PET30 as their DLBCL progressed subsequently. Today, a method to augment predictive power of PET30 is not available.

### Increasing Delta-Liver-SUVmean predicted lasting remission

**Fig. 2:**
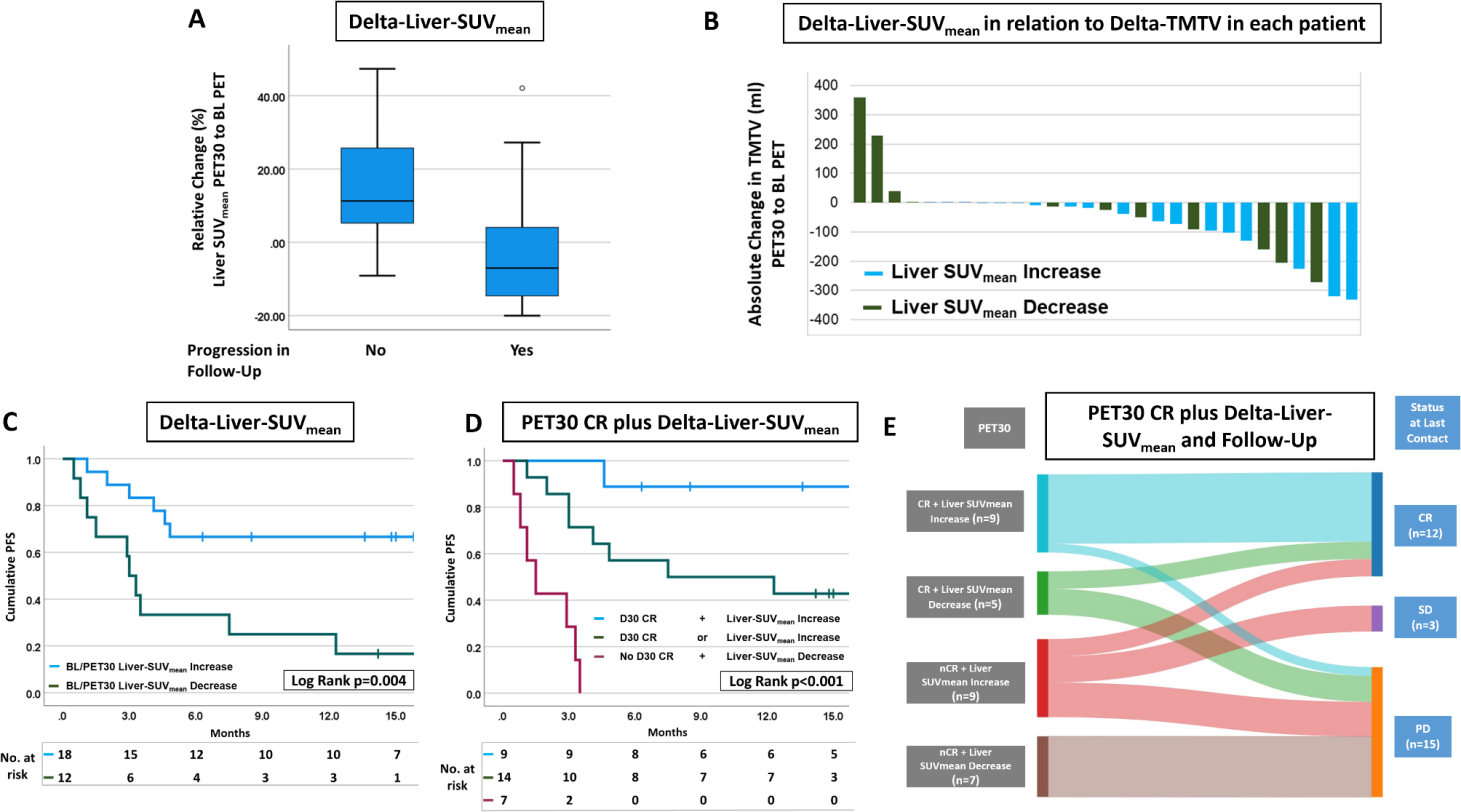
Change of Liver-SUV mean between BL and PET30 predicts progression. **A** Box plot graphic of the percentile change of Liver-SUV_mean_ between BL and PET30 in progressive and non-progressive patients. **B** Bar Chart depicting the absolute change of TMTV between BL and PET30 under CAR-T. Columns are ordered by size with blue color for a patient with an increase in Liver-SUV_mean_ and green color with a decrease in Liver-SUV_mean_. **C** Patient PFS in accordance to increase (blue line) or a decrease (green line) of Liver-SUV_mean_ at PET30 compared with BL. Statistics were done using log-rank test. **D** Kaplan-Meier curves depicting three PFS subgroups generated based on PET30 and Delta-Liver-SUV_mean_. **E** Sankey Diagram as in Fig. 1C depicting the per-patient development of disease remission status from PET30 and Changes of Liver-SUV_mean_ to disease outcome at last contact. PET/CT scan on base line (BL), CD19-CAR-T cell therapy (CAR-T), PET/CT scan at day 30 (PET30), progression-free survival (PFS), standardized uptake values (SUV), total metabolic tumor volume (TMTV)

We then hypothesized that changes in Liver-SUV_mean_ from BL to PET30 may have predictive value for the outcome after CAR-T in patients with relapsed/refractory DLBCL. Over all 30 patients in our cohort, the Delta-Liver-SUV_mean_ increased by a median of + 4.3 ± 18.0% [-1.6-11.8%]. Grouped by remission, median Delta-Liver-SUV_mean_ increased by + 11.3 ± 15.4% [4.9–22.7%] in patients that achieved lasting remission and decreased by -7.0 ± 17.0% [-11.6-6.6%] in patients with disease progression (univariate *p* = 0.010 and multivariate *p* = 0.014; Fig. 2A). The overall availability of FDG in the background organs can be reduced due to a high metabolic activity and thus FDG intake of larger lymphoma masses– a so called FDG-sink effect [31], while a reduced tumor burden can lead to an relative increase in background activity. In our patients though, Delta-Liver-SUV_mean_ and the change of TMTV from BL to PET30 were independent (Spearman-ρ=-0.152, *p* = 0.423, Fig. 2B). In line with their independence, a substantial number of patients with PET30 CR (35.7%) showed a decreasing Liver-SUV_mean_ (Table 3). Furthermore, several patients showed a decrease in FDG-uptake of the liver despite a substantial reduction of metabolic tumor volume strongly arguing against a sink effect (Fig. 2B). Any decrease of Delta-Liver-SUV_mean_ from BL to PET-30 significantly predicted unfavorable PFS (*p* = 0.004, Fig. 2C) with a median PFS of 3.0 [2.3–3.7] months for patients that showed a reduced Delta-Liver-SUV_mean_ from BL to PET-30, while median PFS was not reached in patients with an increase of the Delta-Liver-SUV_mean_. With regard to the CAR-T products used, patients treated with Axi-Cel tended to show a positive Delta-Liver-SUV_mean_ more frequently (13 out of 17) compared to the other CAR-T products which may derive from a slightly higher frequency of responses of 52.9% following Axi-cel compared with 38.5% after all others (*p* = 0.431). However, this difference was not statistically significant in the univariate analysis (*p* = 0.097 / Table 3).

**Table 3:** Patient grouping based on Delta-Liver-SUV_mean_

	Delta-Liver-SUV_mean_		Univariate *p*-values*	Multivariate *p*-values^#^
	Increase (*n* = 18)	Decrease (*n* = 12)		
**CAR-T Product**				
Tisa-Cel Axi-Cel Liso-Cel Experimental CD19	3 13 1 1	5 4 3 0	0.097	0.647
**Delta TMTV (ml)**	-50.4 ± 116.6 [-153.2 - -37.3]	− 19.6 ± 173.8 [-126.2–94.7]	0.232	0.144
**PET30 CR**				
Yes (DS ≤ 3) No (DS ≥ 4)	9 9	5 7	0.654	0.667
**Progression Follow Up**				
Yes No	6 12	10 2	**0.011**	**0.006**
**CRS Grade** (*n*** = 28)**				
≤ 1 2	13 4	9 2	0.654	-
**Delta FDG Uptake Time (Minutes)**	-3.0 ± 11.0 [-6.5–4.8]	-4.5 ± 21.7 [-12.4–15.2]	0.755	0.731
**Delta Blood Sugar (mg/dL)**	1.5 ± 15.1 [-8.9–6.1]	1.5 ± 13.2 [-6.4–10.4]	0.755	0.532

*p-values were determined by Chi-Square test or by Pearson

# CRS Grade was excluded from multivariate analysis due to missing values

Abbreviations: Axicabtagen-Ciloleucel (Axi-Cel), CD19-CAR-T cell therapy (CAR-T), Complete remission (CR), Deauville Score (DS), Cytokine Release Syndrome (CRS), Difference between PET at Baseline and PET at Day 30 (Delta), Fluor-18-Deoxyglucose (FDG), Lisocabtagene Maraleucel (Liso-Cel), PET/CT on Day 30 after CAR-T (PET30), Tisagenlecleucel (Tisa-Cel), Total Metabolic Tumor Volume (TMTV).

Asking, whether Delta-Liver-SUV_mean_ may reflect altered systemic inflammation, we found no correlation with maximum grade of cytokine release syndrome (CRS) after CAR T cell infusion (Spearman-ρ=-0.017; *p* = 0.932) or of the level of C-reactive protein (CRP) (Spearman-ρ=-0.119; *p* = 0.531) or of ferritin (Spearman-ρ=-0.126; *p* = 0.522, Table 3) at lymphodepletion, respectively. Neither blood sugar levels (Spearman-ρ=-0.005; *p* = 0.980) nor time-delay between FDG-injection and start of the PET (Spearman-ρ=-0.082; *p* = 0.668) correlated with Delta-Liver-SUV_mean_. Similar changes, however at a lower, not significant extent, were also found for the blood pool SUV_mean_ (Table 2). Due to the small volume available for the measurement of the blood pool SUV_mean_ and thus a higher dependence of VOI-placement and scanner reconstruction methods with a higher error margin, a more in-depth analysis of this PET parameter was dismissed.

### Estimation of early progression under CAR-T

**Fig. 3:**
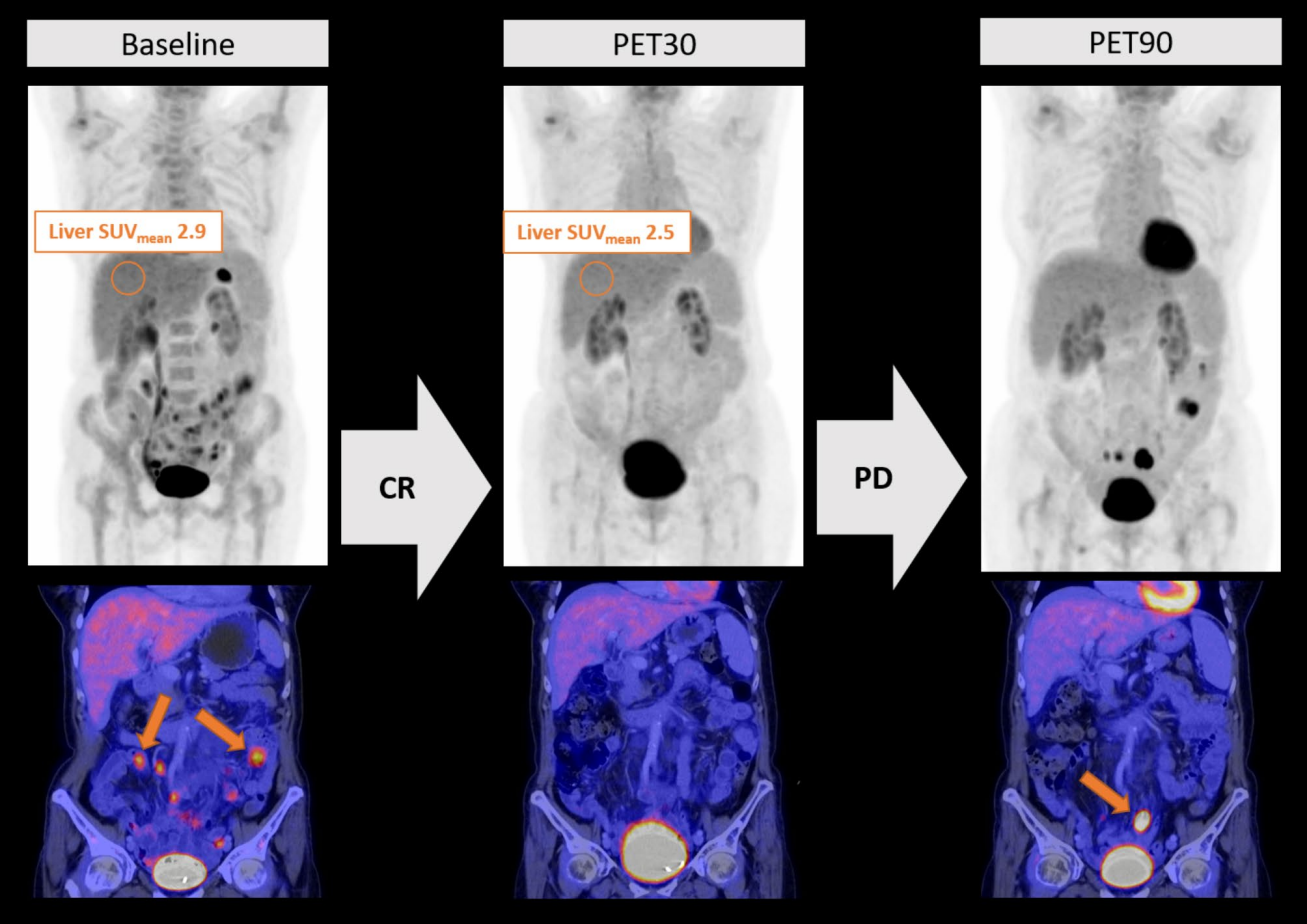
Decrease in Liver-SUV mean is associated with early progression after CAR-T. Representative PET/CT images of in relapse depicting lymphoma at baseline (first column) with multiple FDG-avid intestinal lesions (orange arrows in lower row). PET30 images (second column) demonstrate early metabolic complete remission. Representative measurements of the Liver-SUV_mean_ at Baseline and PET30 are shown in the upper row. PET90 images (third column) depict multiple new intestinal FDG-avid lesions of which the most prominent is marked with an orange arrow in the lower row. CD19-CAR-T cell therapy (CAR-T), Fluor-18-Deoxyglucose (FDG), PET/CT scan at day 30 (PET30), PET/CT scan at day 90 (PET90)

**Fig. 4:**
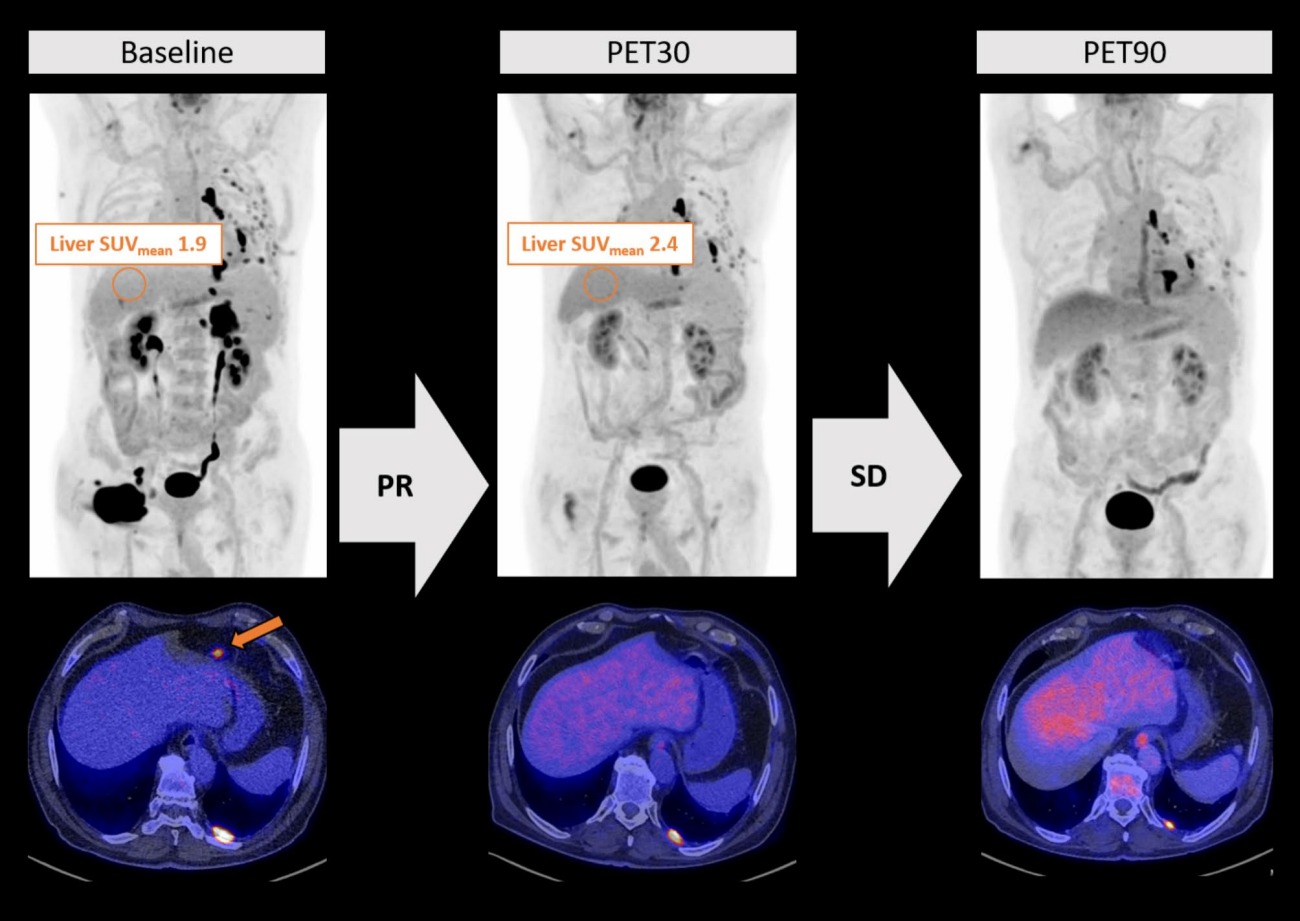
Increase in Liver-SUV mean is associated with longer disease control after CAR-T. Representative PET/CT images of in relapse depicting lymphoma at baseline (first column) with FDG-avid lymphonodale manifestations in the abdomen and the right inguinal region. Furthermore PET/CT presents with highly metabolic active pleural lesions of unclear origin mostly associated with pleurodesis but lymphoma could not be ruled out entirely. PET30 after CAR-T (second column) shows decreasing metabolic activity of the lymphoma with remaining FDG-avid pleural lesions overall rated as partial remission. Representative measurements of the Liver-SUV_mean_ at Baseline and PET30 are shown in the upper row. PET90 images (third column) depicts stable disease with multiple FDG-avid pleural lesions. Two years after CAR-T the patients remains in disease remission. CD19-CAR-T cell therapy (CAR-T), Fluor-18-Deoxyglucose (FDG), PET/CT scan at day 30 (PET30), PET/CT scan at day 90 (PET90)

We used a functional linear cox regression model to estimate the predictive value of LDH at LD, PET30-CR and Delta-Liver-SUV_mean_ for progression under CAR-T. Both PET30-nCR (*p* = 0.001; hazard ratio (HR) 4.79 [2.3–33.6]) and decrease of Delta-liver-SUV_mean_ (*p* = 0.002; HR 3.99; [2.1–23.1]) were significantly associated with a poor outcome, while LDH at LD remained not significantly predictive in multivariate analysis (*p* = 0.191; HR 2.36 [0.7–8.5]). Adding Delta-Liver-SUV_mean_ enhanced PET30-CR in predicting outcome (Fig. 2D). Of the 9 patients with PET30-CR and increasing Delta-Liver-SUV_mean_ only one patient relapsed (good-risk group; Fig. 2E). All seven patients with PET30-nCR and decreasing Delta-Liver-SUV_mean_ relapsed within less than 4 months (very high-risk group, Fig. 2E). The combined group of patients for whom PET30 and Delta-Liver-SUV_mean_ showed either nCR and an increase or CR and a decrease showed an intermediate PFS of in median 7.5 [0.0-21.2] months (Fig. 2D). A more detailed analysis of this intermediate group showed 3/5 patients (60%) with CR in PET30 and decreasing Delta-Liver-SUV_mean_ progressed in follow-up, despite excellent initial response in PET30 (Fig. 2D). A representative case can be found in Fig. 3. On the other hand, 5/9 patients with metabolically active lesions in PET30 and increasing Delta-Liver-SUV_mean_ remained in disease remission long-term. All seven patients with metabolic activity in PET30 and decreasing Delta-Liver-SUV_mean_ relapsed within 4 months after CAR-T (Figs. 2E and 4).

## Discussion

Offering the possibility of early intervention following CAR-T in aggressive lymphoma, PET30 has recently evolved as a strong predictor for lasting remission [20, 32, 33]. However, high rates of false-positive and false-negative prediction by PET30 remain problematic when justifying additional treatment in imminent relapse. Here we show that incorporation of Delta-Liver-SUV_mean_ to PET30 enabled a more accurate differentiation between the poorest and the most favorable responses with the possibility to guide early interventions post CAR-T. Additionally, we identified a group of patients with intermediate PFS. Although limited by the relatively small patient number, we found that patients with a metabolic CR in PET30 had an increased risk of relapse if Delta-Liver-SUV_mean_ decreased, warranting an intensified clinical monitoring for these patients. Though increase of Delta-Liver-SUV_mean_ was found to be a positive predictive biomarker in interim PET scans in a study of Hodgkin’s lymphoma undergoing treatment with Adriamycin, Bleomycin, Vinblastine and Dacarbazine (ABVD) [22], the possible additive value of increased Delta-Liver-SUV_mean_ in PET30 compared with BL to predict responses to CAR-T cell therapy identified herein has not been described before. Derlin et al. identified an increased FDG-uptake in immunologically active organs at PET30 in a small cohort of DLBCL patients responding to CAR-T as a possible biomarker associated with favorable PFS [8, 22]. In line with reduced FDG-uptake of the liver, they found that a reduced FDG-uptake in the spleen and lymph nodes was associated with poorer outcome [8]. Delta-Liver-SUV_mean_ was not discussed by Derlin et al. In line with our data, these findings were independent of CRS or Immune Effector Cell Associated Neurotoxicity (ICANS). The authors hypothesize that the decreased metabolic activity of spleen and lymph nodes are due to a lack of systemic inflammation which they interpreted as a possible sign of missing CAR-T expansion. The liver is central to inflammatory processes and produces a vast number of acute phase proteins [34, 35]. Correlation of liver inflammation and increased FDG uptake is supported by heightened uptake in active, checkpoint inhibitor-induced hepatitis and by a link between FDG-uptake and inflammation-associated liver alterations [36, 37]. Elevated acute phase proteins in the serum of CAR-T cell patients correlate with higher rates of ICANS and CRS and with higher efficacy [38–41]. As such, elevated acute phase may argue towards the liver uptake as a possible predictor of CAR T cell efficacy and thus, lasting remission [8, 29, 42]. That change in liver SUV_mean_ might represent systemic inflammation is further supported by the similar results in the blood pool SUV_mean_ which however did not reach significance (Table 2). In contrast, a larger tumor in progression could take up most of the infused FDG and as such reduce FDG-uptake of the liver passively - a so called FDG-sink effect [31], while, a reduced tumor burden resulting in relatively lower FDG-consumption by the tumor mass could then lead to an increased FDG utilization in the reference organs like the liver. That Delta-Liver-SUV_mean_ keeps decreasing in some patients despite substantial responses to CAR-T with up to 280 mL of TMTV reduction (Fig. 2B) supports a reason other than sink effect for the described reduced FDG-uptake of the liver and is distinct from the interpretation of interim PET results from studies in Hodgkin’s lymphoma under ABVD therapy [43]. Beyond a sink effect, we could also not identify a bias in other critical values including blood sugar levels or time from FDG-injection to imaging. Due to numerous possible factors that could influence Liver-SUV_mean_, our findings must be interpreted with care and will have to be prospectively validated in a larger patient cohort. However, that immune status and the tumor microenvironment is tightly linked to CAR-T efficacy has repeatedly been implicated and studies measuring not only the metabolic activity of tumor lesions but also the metabolic response of immunologically active organs may substantially enhance response interpretation by PET in the future [8, 15, 16, 29, 33, 44]. The subtle changes of physiological FDG-uptake patterns, which might be missed by human readers also argue towards the implantation of radiomics and deep learning models. The individual CAR-T products all used CD-19 as the target antigen. However, differences exist in the co-stimulatory endodomain, with CD-137 for Tisa-Cel and Liso-Cel, and CD-28 for Axi-Cel, which also regulates the immunological reshponse and thus could differently influence the metabolic reaction in lymphatic organs and the CAR-T cell expansion. Hence, a potential bias regarding Delta- Liver-SUV_mean_ cannot be dismissed. In our patient cohort, there was a tendency for a stronger increase in Delta-Liver-SUV_mean_ in PET30 in the group of patients treated with Axi-Cel. However, the differences between the groups were not significant (Table 3). This might be due to the small cohort size but could also correlate with the higher rates of durable remissions achieved with Axi-cel and is thus in line with our novel biomarker of response. Here too, further prospective studies are necessary to more precisely evaluate the influence of the individual CAR-T products on metabolic measurements of the liver in PET30. In line with previous studies [10], LDH levels were significantly higher in the group of patients with poor response to CAR-T therapy. Interestingly, the prognostic value could not be confirmed in the functional linear cox regression analysis. This is also most likely due to the small number of patients.

The relatively small cohort size and its retrospective nature are the major limitations of the study. Because PET30 has not routinely been used in many CAR T cell centers, we could not identify a reasonably sized control cohort leaving our data unvalidated at this time. Despite these limitations, we are the first to report on longitudinal changes in Liver-SUV_mean_ as possibly predictive towards PFS of DLBCL patients following CAR-T.

## Conclusion

PET30-CR is associated with a good response to CAR-T, however high rates of false-positive and false-negative remain problematic. Additionally to PET30 metabolic response, longitudinal metabolic changes in Liver-SUV_mean_ predicted CAR-T efficiency. Our enhanced prediction could inform future early intervention studies with the overall-goal of improved long-term outcome following CAR-T.

## Electronic supplementary material

Below is the link to the electronic supplementary material.

Supplementary Material 1

## Data Availability

The datasets generated during and/or analyzed during the current study are available from the corresponding author on reasonable request.

## Abbreviations

Axi-Cel: Cel-Axicabtagen-Ciloleucel
BL: Baseline PET/CT prior to CAR-T
CAR: T-CD19-CAR-T cell therapy
CR: Complete Metabolic Remission
CRP: C-reactive protein
CRS: Cytokine Release Syndrome
Delta: Difference between BL and PET30
DLBCL: Diffuse large B cell Lymphoma
DS: Deauville Score
ECOG: Eastern Cooperative Oncology Group Performance Status
FDG: Fluor-18-Deoxyglucose
ICANS: Immune Effector Cell Associated Neurotoxicity
IMPI: International Metabolic Prognostic Index
nCR: Incomplete Metabolic Remission
R-IPI: Revised International Prognostic Index
LD: Lymphodepletion
LDH: Lactate dehydrogenase
Liso-Cel: Cel-Lisocabtagene Maraleucel
MTV: Metabolic Tumor Volume
PET: Positron Emission Computed Tomography
PET30: PET/CT on Day 30 after CAR-T
PET90: PET/CT on Day 90 after CAR-T
PFS: Progression-free Survival
SUV: Standardized Uptake Values
tFL: transformed follicular lymphoma
Tisa-Cel: Cel-Tisagenlecleucel
TMTV: Total Metabolic Tumor Volume
VOI: Volume of Interest
