# Supplementary figures and images for: Liver-FDG-uptake augments early PET/CT prognostic value for CD19-targeted CAR-T cell therapy in diffuse large B cell lymphoma
Source: EJNMMI Res. 2025 Mar 17;15:25. doi: 10.1186/s13550-025-01201-1 (PMC11914545; doi:10.1186/s13550-025-01201-1)

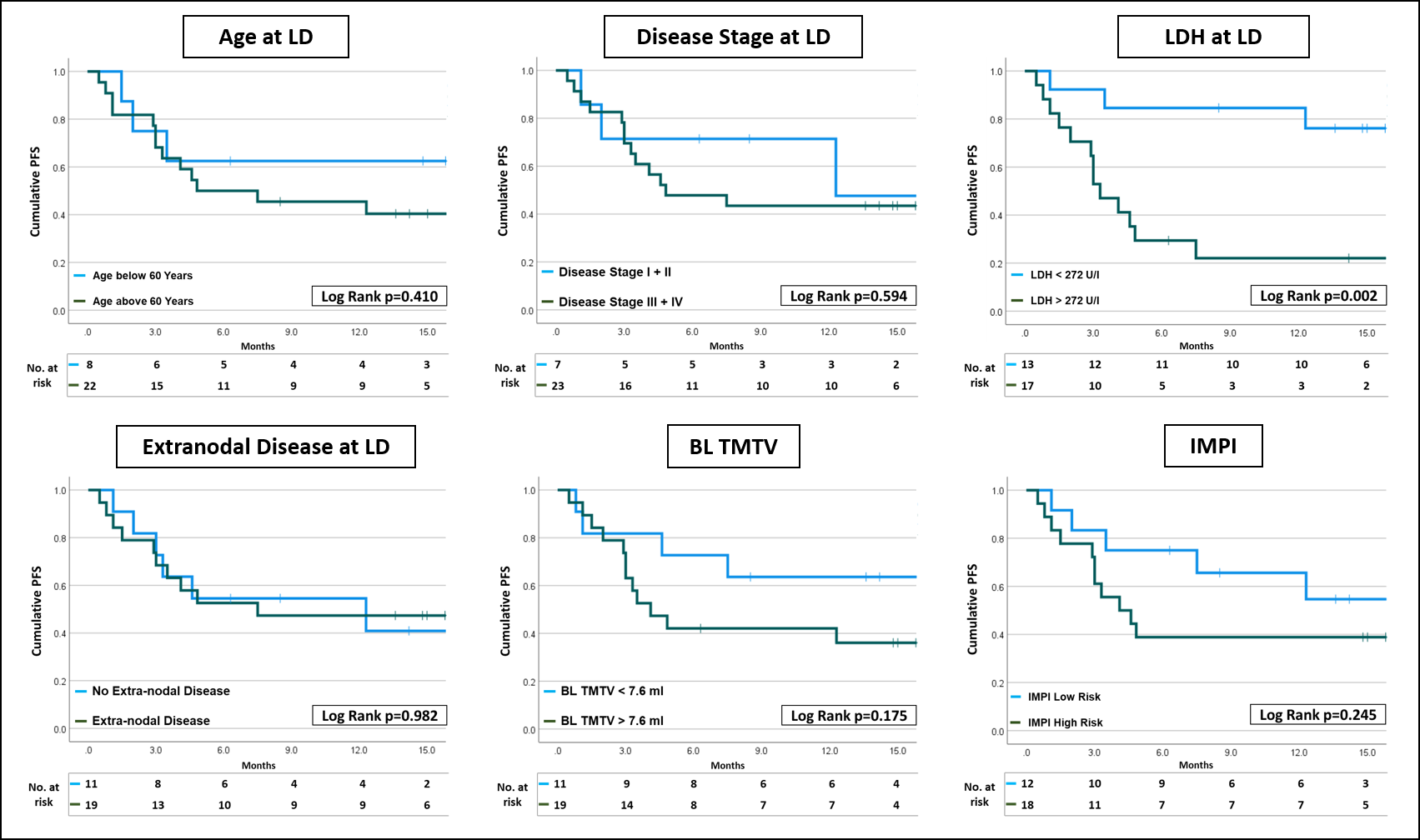

Supplement: Supplementary file 1 — Supplementary Material 1 [file 13550_2025_1201_MOESM1_ESM.png]
